# Clinical characteristics and prognosis of ventricular primary central nervous system lymphoma: a case series and literature review

**DOI:** 10.1186/s41016-025-00410-w

**Published:** 2025-10-10

**Authors:** Chenqi He, Nian Jiang, Zujian Xiong, Abraham Ayodeji Adegboro, Siyi Wanggou, Xuejun Li

**Affiliations:** 1 Department of Neurosurgery, Xiangya Hospital, Central South University, Changsha, Hunan 410008 P. R. China; 2 Hunan International Scientific and Technological Cooperation Base of Brain Tumor Research, Xiangya Hospital, Central South University, Changsha, Hunan 410008 P. R. China

**Keywords:** Primary central nervous system lymphoma, Clinical features, Prognosis

## Abstract

**Background:**

Primary central nervous system lymphoma (PCNSL) is a rare intracranial tumor, and even rarer when the lesion involves the ventricles. This article aims to evaluate the clinical features of PCNSL lesions involving the ventricles.

**Methods:**

Gathering data from a single institution, we conducted a retrospective analysis of all patients with pathologically proven PCNSL between August 2004 and August 2021 from Xiangya Hospital databases and searched for previously reported cases in PubMed. The MRI signal characteristics, location, size, and hydrocephalus presence were evaluated on the images.

**Results:**

We identified 29 new cases and reviewed 22 previously reported cases. The study included 32 (62.75%) males and 19 (37.25%) females, with a median age of 53 years. Of the 22 cases with preoperative imaging available for review, all lesions appeared as isointense or hyperintense on T1WI. Twenty lesions presented as isointense or hypointense, and two cases presented as hyperintense on T2WI. The median Ki-67 of PCNSL involving the ventricle was 80%. BCL-2 was positive in 10/22 (45.45%) cases, and BCL-6 was positive in 18/28 (64.29%) cases.

**Conclusions:**

We found that age ≥ 60 years old and female gender were significant risk factors for survival outcome (*P* < 0.05). In terms of imaging presentation and prognosis, PCNSL lesions invading the ventricles were similar to those that were non-invasive. Our findings further suggest that ventricular PCNSL may have more diverse signal types on T2WI. Therefore, we recommend that ventricular lymphoma be treated in the same way as non-ventricular PCNSL.

## Background

Primary central nervous system lymphoma (PCNSL) is a rare and highly aggressive non-Hodgkin’s lymphoma confined to the central nervous system, including the brain, spine, cerebrospinal fluid, and eyes, without other systems being involved [1]. In the past three decades, the incidence of PCNSL has increased from 1% to 3–4% of all newly diagnosed primary brain tumors [2, 3]. Despite recent improvements in treatment strategies, the prognosis of PCNSL remains unsatisfactory, with 5-year survival rates of only 30 to 40% [4]. The disease can theoretically arise from all types of pathology, with diffuse large B-cell lymphoma (DLBCL) being the most common type, accounting for approximately 90% of cases, while Burkitt and T-cell lymphoma are rare [5]. PCNSL is typically located in various regions of the brain, including the frontal lobe, temporal lobe, parietal lobe, occipital lobe, basal ganglia, periventricular brain parenchyma, and corpus callosum. However, ventricular involvement is a rare occurrence [6, 7], and full knowledge of the histologic findings, radiologic features, and therapeutic methods is currently limited to case reports.

To expand our understanding of PCNSL involving ventricles, we present 29 newly diagnosed cases from Xiangya Hospital and conducted a retrospective analysis of previously reported cases in the PubMed database. Our search yielded 22 cases of PCNSL involving ventricles reported in English [8–29], bringing the total number of cases to 51. Through this retrospective evaluation, we aimed to analyze the clinical characteristics, therapeutic methods, pathological features, and radiological presentations of PCNSL involving ventricles, with the goal of evaluating their effect on outcomes and developing optimal treatment regimens.

## Methods

### Patients and study design

This retrospective study was approved by the Human Research Ethics Committee of Xiangya Hospital Central South University (No. 202202034). The requirement for informed consent was waived by the Ethics Committee due to the retrospective nature of the study. The patients’ prognoses were assessed using clinical follow-up and telephone interviews. Subjects enrolled in this cohort were recruited from among the hospitalized patients of the neurosurgery department of Xiangya Hospital between August 2004 and August 2021.

**Fig. 1:**
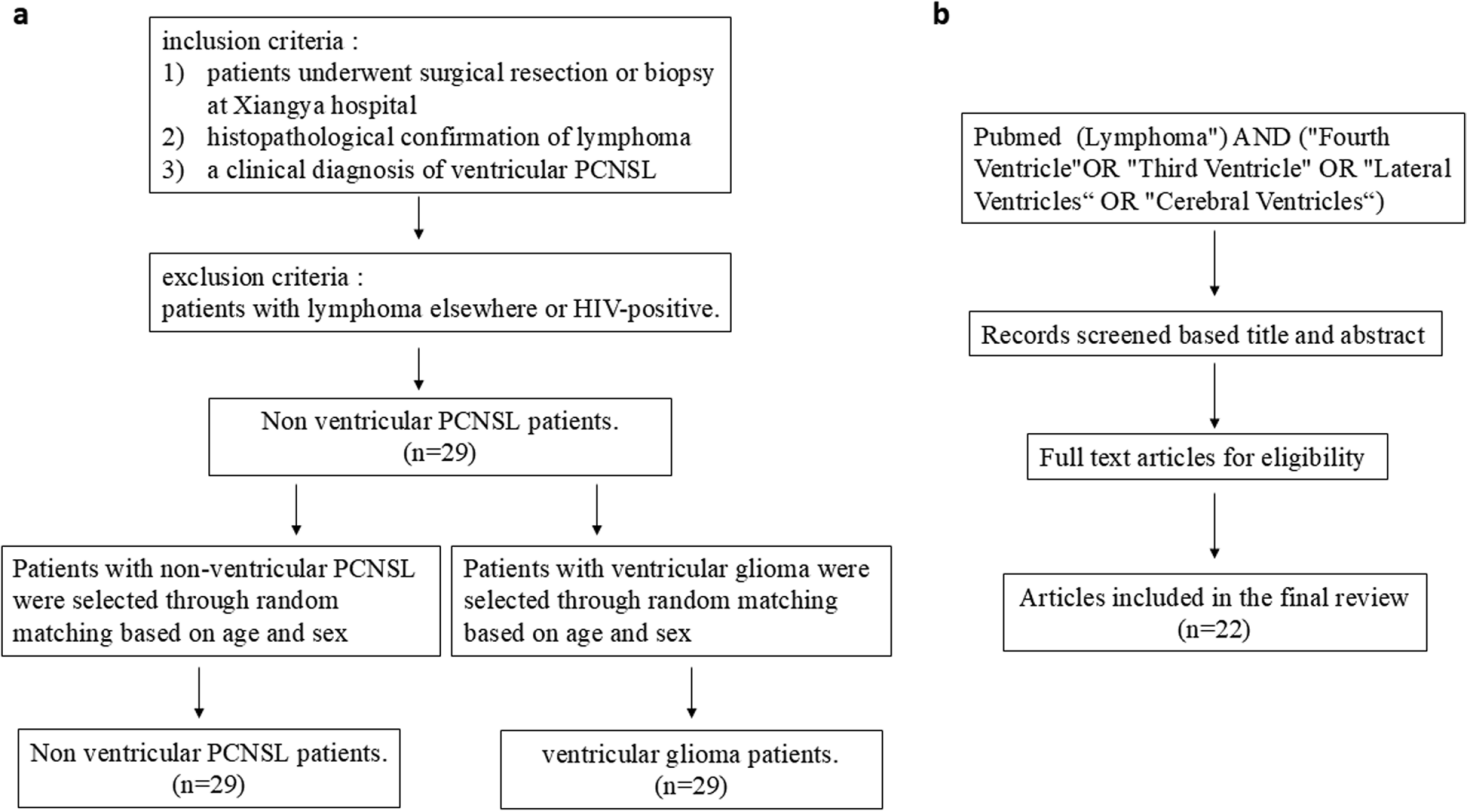
Study design and literature review process. Flowchart of patient selection (**a**). Literature search and screening strategy (**b**)

The authors applied the following inclusion criteria: (1) patients who underwent surgical resection or biopsy at Xiangya Hospital, (2) histopathological confirmation of lymphoma, and (3) a clinical diagnosis of ventricular PCNSL. Patients were excluded if they met any of the following criteria: (1) presence of systemic lymphoma or (2) HIV-positive status (Fig. 1). As for the location, the term “involving ventricle” means that the tumor was located in the ventricles with or without the brain parenchyma. Simultaneously, we searched the PubMed database to find PCNSL involving ventricle using the following search terms: ("Lymphoma"[Mesh]) AND ("Fourth Ventricle"[Mesh] OR "Third Ventricle"[Mesh] OR "Lateral Ventricles"[Mesh] OR "Cerebral Ventricles"[Mesh]). Ultimately, 29 new cases and 22 previously published cases of PCNSL constituted the study cohort (Tables 1 and 2).

**Table 1:** Published cases of PCNSL involving ventricle

Case	Sex	Age	Clinical characteristics	Involved ventricles	Treatment
1 [28]	M	71	1-month history of vertigo, vomiting and weight loss	4 V and lateralV	CTx
2 [27]	M	6	8-day of paroxysmal headache	4 V	Operation and CTx
3 [26]	M	65	2-week of weight loss, headaches, blurred vision, asthenia and quickly walking impairment	4 V	Immunotherapy
4 [25]	M	66	2-month of dizziness and 1-month of diplopia	4 V and lateralV	Operation exploration and CTx
5 [24]	M	77	1-week of intermittent vertigo, nausea, vomiting, and progressively unsteady gait	4 V	Operation total resection
6 [23]	F	60	3-month of diplopia	4 V	Operation total resection and CTx
7 [22]	M	59	8-month of vomiting and vertigo	4 V	Operation total resection
8 [21]	M	27	10-day of nausea, vomiting, and headache	lateralV	CTx and immunotherapy
9 [21]	F	31	Headaches, vision difficulties and ataxia	lateralV	Operation, CTx, RTx, and immunotherapy
10 [20]	F	63	Seizure then progressively worsening headaches and dizziness for 1 week	3 V, 4 V and lateralV	Operation
11 [19]	F	65	4-week of intractable vomiting and headaches	4 V, lateralV	CTx
12 [18]	M	62	15-day vomiting and mild preceding nausea	3 V, 4 V and lateralV	CTx and RTx
13 [17]	M	15	6-month of headache, vomiting, and seizure	4 V and lateralV	Operation biopsy
14 [16]	F	44	Acute episode of headache with hyperpyrexia	lateralV	Operation partial resection
15 [15]	F	75	2-week of intermittent speech disturbance, headache, and general malaise	3 V and lateralV	Operation total resection and RTx
16 [14]	M	71	1-month of general fatigue	3 V	RTx
17 [13]	M	63	Transient monoparesis of the left leg	lateralV	Operation total resection and CTx
18 [12]	F	55	2-week of progressive deterioration of memory, dysarthria, and urinary incontinence	lateralV	RTx
19 [11]	M	69	6-week history of spontaneous unrelenting vomiting, with mild preceding nausea	4 V	CTx
20 [10]	M	53	Persistent headaches and a seizure	lateralV	Operation total resection and CTx
21 [9]	F	57	1-week of progressive visual deterioration	3 V	Operation biopsy and CTx
22 [8]	F	33	4-week of vertigo and headaches	4 V	Operation total resection, CTx, RTx, and ASCT

*M* male, *F* female *3, V* the third ventricle, *4 V *the fourth ventricle, *lateralV* the lateral ventricle, *CTx* chemotherapy, *RTx* radiotherapy

**Table 2:** Summary of 29 patients with PCNSL involving ventricles

Case	sex	Age (year)	Category	Involved ventricle	tumor size (cm)	Lesion number	T1WI	T2WI	T2WI mixed signal	Enhancement	Hydrocephalus before operation	OS (months)
1	M	6	/	3 V and lateralV	9.7	3	Hypo	Hypo	Yes	Yes	No	0
2	F	18	GCB	4 V and lateralV	5.31	3	Hypo	Iso	Yes	Yes	Yes	/
3	M	27	GCB	3 V, 4 V, and lateralV	3.2	4	Iso	Iso	No	Yes	Yes	/
4	F	27	GCB	4 V	/	/	/	/	/	/	/	120 +
5	M	28	GCB	3 V and lateralV	3	2	Iso	Iso	Yes	Yes	No	11 +
6	M	40	Non-GCB	4 V and lateralV	2.3	2	Hypo	Iso	Yes	Yes	No	10
7	M	45	GCB	3 V	2.25	1	Hypo	Hypo	No	Yes	Yes	/
8	M	45	Non-GCB	lateralV	/	/	/		/	/	/	32 +
9	M	46	/	3 V	3.42	1	Hypo	Iso	Yes	Yes	Yes	1
10	M	47	Non-GCB	4 V and lateralV	8.88	3	Hypo	Hypo	No	Yes	No	/
11	M	47	Non-GCB	lateralV	6.34	1	Iso	Hypo	Yes	Yes	No	/
12	M	49	Non-GCB	lateralV	9.23	2	Hypo	Hypo	Yes	Yes	No	71 +
13	M	51	Non-GCB	lateralV	1.9	1	Hypo	Iso	No	Yes	Yes	/
14	M	52	/	lateralV	7.44	1	Hypo	Hypo	No	Yes	Yes	71 +
15	F	52	GCB	lateralV	/	/	/	/	/	/	/	0
16	M	52	Non-GCB	lateralV	7.1	2	Hypo	Iso	Yes	Yes	No	10
17	F	53	GCB	lateralV	3.45	1	Hypo	Hypo	Yes	Yes	No	3
18	M	53	GCB	4 V and lateralV	/	2	Hypo	Iso	Yes	Yes	No	21 +
19	M	53	GCB	lateralV	4.1	1	Hypo	Hypo	Yes	Yes	No	/
20	M	54	Non-GCB	lateralV	3.89	1	Hypo	Hypo	Yes	Yes	No	21 +
21	M	56	Non-GCB	lateralV	7.88	1	Hypo	Iso	No	Yes	No	17
22	M	59	Non-GCB	lateralV	5.65	1	Hypo	Hyper	Yes	Yes	No	46 +
23	M	59	Non-GCB	lateralV	4.18	1	Hypo	Iso	Yes	Yes	No	12
24	F	59	Non-GCB	lateralV	5.11	1	Hypo	Hyper	Yes	Yes	No	9
25	F	60	Non-GCB	lateralV	/	/	/	/	/	/	/	10
26	F	60	Non-GCB	4 V	2.16	1	Hypo	Iso	Yes	Yes	No	5 +
27	F	62	GCB	lateralV	/	/	/	/	/	/	/	3
28	F	63	GCB	lateralV	/	/	/	/	/	/	/	9
29	F	68	GCB	4 V	/	/	/	/	/	/	/	9

*M* male, *F* female, *3 V* the third ventricle, *4 V* the fourth ventricle, *lateralV* the lateral ventricle

Two matched groups were ultimately created by matching the sex and age of PCNSL cases. One group was for PCNSL not involving the ventricles and the other was for ventricular gliomas. Given the incomplete information of old PubMed cases, we collected only 29 patients as the controls in our study. We analyzed the differences in the radiological features, pathological characteristics, clinical symptoms, perioperative complications, treatments, and prognoses between PCNSL involving ventricles and those not involving ventricles. Meanwhile, the perioperative complications and clinical characteristics were compared in PCNSL involving ventricles and glioma involving ventricles. Patients’ characteristics, including demographics, clinical symptoms, imaging data, pathological results, perioperative complications, and treatments, were recorded in detail. Individual patients may present with one or more clinical symptoms concurrently. The performance status of a patient prior to an operation was assessed using Eastern Cooperative Oncology Group (ECOG) scores [29]. An ECOG score below 3 was considered to be indicative of a patient’s good health status. The treatment was divided into surgery and biopsy, ignoring whether it was gross total resection or subtotal resection. Chemotherapy and radiotherapy were included in nonsurgical treatment, ignoring the type and dose of the medicine. Furthermore, the perioperative complications were faithfully recorded, such as intracranial infection, pulmonary infection, vein thrombosis, hydrocephalus, and intracranial hemorrhage. The survival time of patients was provided by themselves or their relatives via a telephone interview or outpatient recording. The primary prognostic measure was overall survival (OS), the survival time and survival status (living or dead).

### Neuroimaging

In this study, we scrutinized all scans of patients with PCNSL involving ventricles to investigate the location, size, and signal characteristics of the neoplasms. We also meticulously recorded perilesional edema, enhancement characteristics, calcifications, and hemorrhage. However, due to some patients undergoing scans at other institutions, not all patients received CT or MRI scans before surgery at our institution. Out of the total patients, 10 received preoperative CT scans, and 22 patients received preoperative MRI scans at Xiangya Hospital.

### Pathology

To obtain pathology information, we utilized the medical records system to collect data on immunohistochemistry results, including GFAP, MUM-1, BCL-2, BCL-6, CD10, CD20, CD3, CD5, EBER, Ki-67, and other relevant factors. The final diagnoses were confirmed by two independent neuropathologists using the WHO’s classification, based on both morphological and immunohistochemical analyses. BCL-2, BCL-6, and Ki-67 have been considered to be prognostic factors for DLBCL [30–32]. EBER is utilized to exclude Epstein-Barr (EB) virus infection.

DLBCL is classified into germinal center B-cell (GCB) and non-GCB categories based on immunohistochemical results for CD10, BCL-6, and MUM1. Tumors with CD10 (−), BCL-6 (+ or −) and MUM-1 (−) as well as tumors with CD10 (+) were categorized as GCB subtypes. Tumors with MUM-1 (+) and CD10 (−), regardless of BCL-6 (+ or −) were classified as non-GCB subtypes [33].

### Analyzed data and statistics

All statistical analyses were performed using IBM SPSS 25.0 for Windows. The data were reported as the mean ± standard deviation when the data were normally distributed. Non-normally distributed data were represented by the median (P25, P75). The Wilcoxon rank sum and the *χ*^2^ tests were used for univariate analysis. The Kaplan–Meier method and log-rank test were used to perform survival analysis. P < 0.05 was considered to be statistically significant. Due to limited sample size, survival analyses were conducted using univariate Kaplan–Meier methods without multivariate adjustment.

## Results

### Clinical Data

In this study, a total of 51 cases of PCNSL involving ventricles were analyzed, including 22 previously reported cases and 29 new cases.

Among the 29 cases from Xiangya Hospital, the median age was 52 years (range: 6–68 years), including 19 males and 10 females. Among the 22 previously published cases, the median age was 61 years (range: 6–77 years), with 13 males and 9 females (Tables 2 and 3). A Mann–Whitney *U* test revealed a statistically significant difference in age distribution between the two cohorts (*P* = 0.025). However, a chi-square test showed no significant difference in sex distribution between the groups (*P* = 0.638).

**Table 3:** Comparison of PCNSL involving ventricles and PCNSL not involving ventricles

Factors		PCNSL involving ventricles	PCNSL not involving ventricles	P value	95% CI
Age (year) median (P25, P75)		52(45, 59)	51(42, 55)	0.513	− 4.000, 7.000
Sex (*n*, %)	Male	19 (65.52)	19 (65.52)	1.000	0.339, 2.953
	Female	10 (34.48)	10 (34.48)		
ECOG (*n*, %)	0–2	18 (62.07)	23 (79.31)	0.248	0.132, 1.376
	3–5	11 (37.93)	6 (0.21)		
Treatment (*n*, %)	Surgery	27 (93.10)	22 (75.86)	NA	
	Biopsy	2 (6.90)	7 (24.14)		
Chemotherapy (*n*, %)	Yes	17	19	NA	
	No	5	7	NA	
Radiotherapy (*n*, %)	Yes	7	9	NA	
	No	11	16	NA	
Ki-67 (%) median (P25, P75)		80 (61.25, 90)	80 (70, 90)	0.399	10.000, 0.000
BCL-6 (*n*, %)	** − **	10 (35.71)	3 (10.34)	0.022	1.160, 19.985
	** + **	18 (64.29)	26 (89.65)		
BCL-2 (*n*, %)	**-**	12 (54.55)	7 (28.00)	0.081	0.919, 10.358
	** + **	10 (45.45)	18 (72.00)		

All patients were HIV-negative and presented with A symptoms. The clinical manifestations of PCNSL involving ventricles were found to be different from those not involving ventricles. Specifically, 24 (47%) PCNSL patients involving ventricle had a headache, while only 16 (55%) PCNSL patients without ventricular involvement had a headache (Table 4). In addition, dizziness, vomiting (and/or nausea), vision impairment, memory impairment, and limb weakness were common symptoms (accounting for over 16% of cases) in patients with ventricular involvement.

**Table 4:** Clinical manifestations of the three diseases

Clinical manifestations	PCNSL involving ventricles (*n* = 29)	PCNSL not involving ventricles (*n* = 29)	Glioma involving ventricles (*n* = 29)
Headache	12	16	17
Dizzy or vertigo	9	9	10
Vomiting (and/or nausea)	5	7	10
Vision impairment/loss	3	3	1
Memory impairment	9	1	6
Limb weakness/walking impairment	4	10	3
Seizure	1	0	2
Fatigue	1	0	1
Polyuria	3	0	0
Speech disturbance	1	0	1
Consciousness disorder	2	0	0
Tinnitus	1	1	0

The table does not list all the clinical symptoms; it omits those that occurred only once

### Radiological features

Among the 29 newly reported cases of PCNSL involving ventricles, 10 patients underwent preoperative CT scans, which showed a hyperintense or isointense signal. On the other hand, 22 patients underwent preoperative MRI, with 13 patients having one lesion, 5 having two lesions, 3 having three lesions, and 1 having four lesions (Table 2). Multiple lesions refer to tumor involvement in different ventricular compartments. On T1WI, all lesions were hypointense or isointense. However, on T2WI, twenty lesions were isointense or hypointense, with only two lesions being hyperintense relative to gray matter. MRI revealed mixed signals in 25 lesions (69.44%) on T2WI.

Of the 22 cases, all demonstrated enhancement and edema, and none had necrosis, cystic degeneration, or calcification. The median size of the tumor was 5.21 cm in diameter, ranging from 1.90 cm to 9.70 cm. Six patients had hydrocephalus because of extensive contiguity with ventricular structures and large lesion size.

**Fig. 2:**
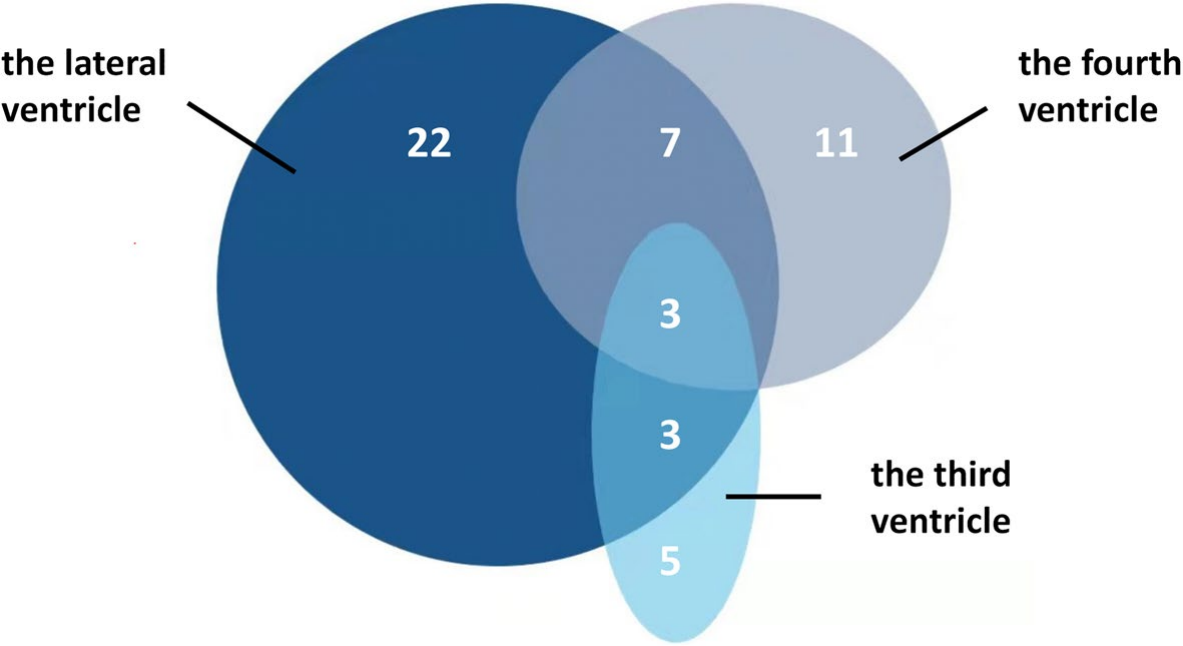
The distribution diagram of 59 cases of PCNSL involving ventricles, and one case can involve single or multiple ventricles

**Fig. 3:**
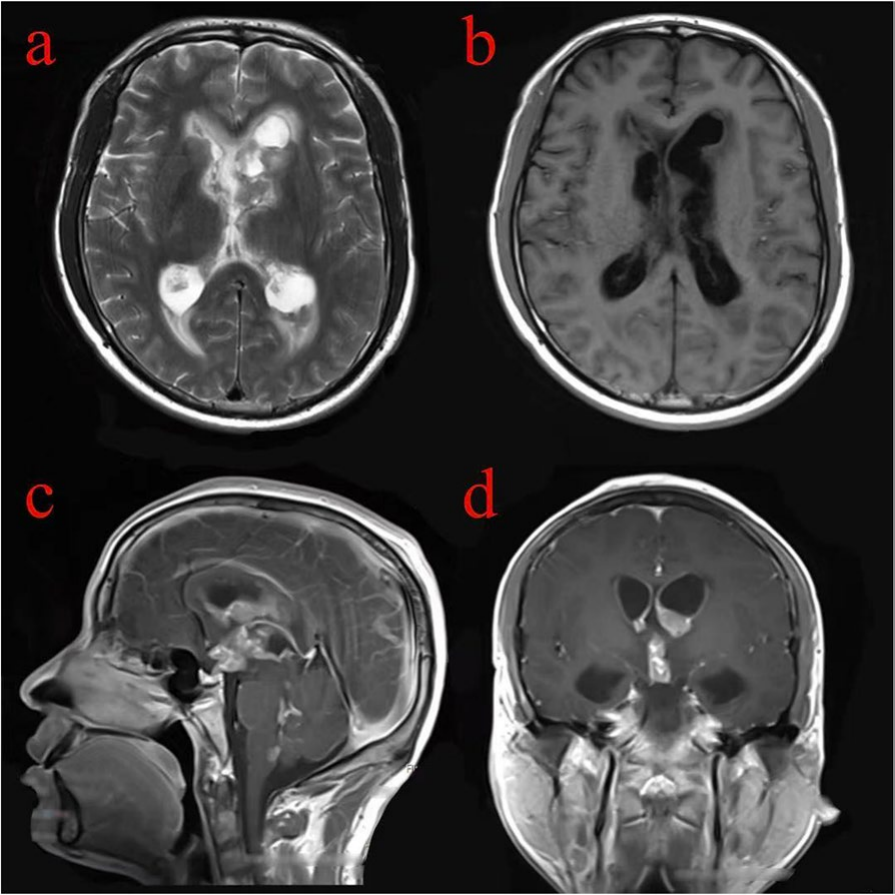
Lesions involving all of the ventricles. Mixed hypointense signal on axial T2WI (**a**). Hypointense signal on axial T1WI (**b**). Sagittal and coronal T1 sequence with gadolinium contrast demonstrates enhancing lesions affecting all of the ventricles (**c** and **d**)

**Fig. 4:**
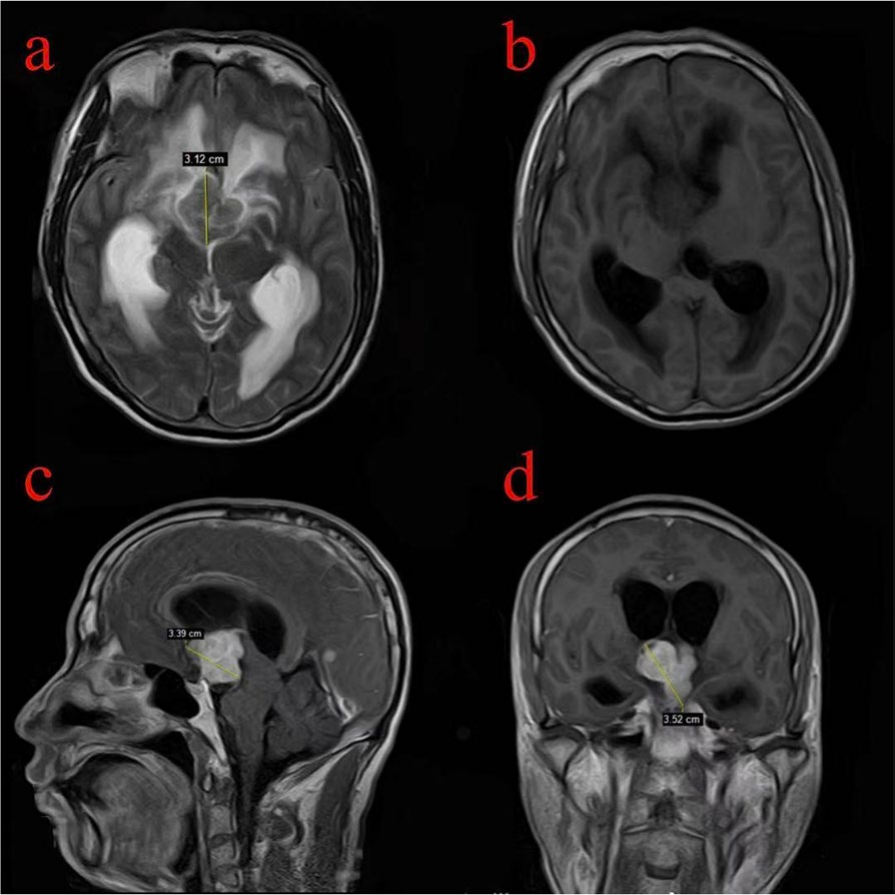
The lesion located in the third ventricle displayed an isointense signal on axial T2WI (**a**) and a hypointense signal in the third ventricle on axial T1WI (**b**). Sagittal and coronal T1c demonstrate enhancing lesions in the third ventricle (**c** and **d**). The maximum tumor diameter is 3.52 cm (**d**)

**Fig. 5:**
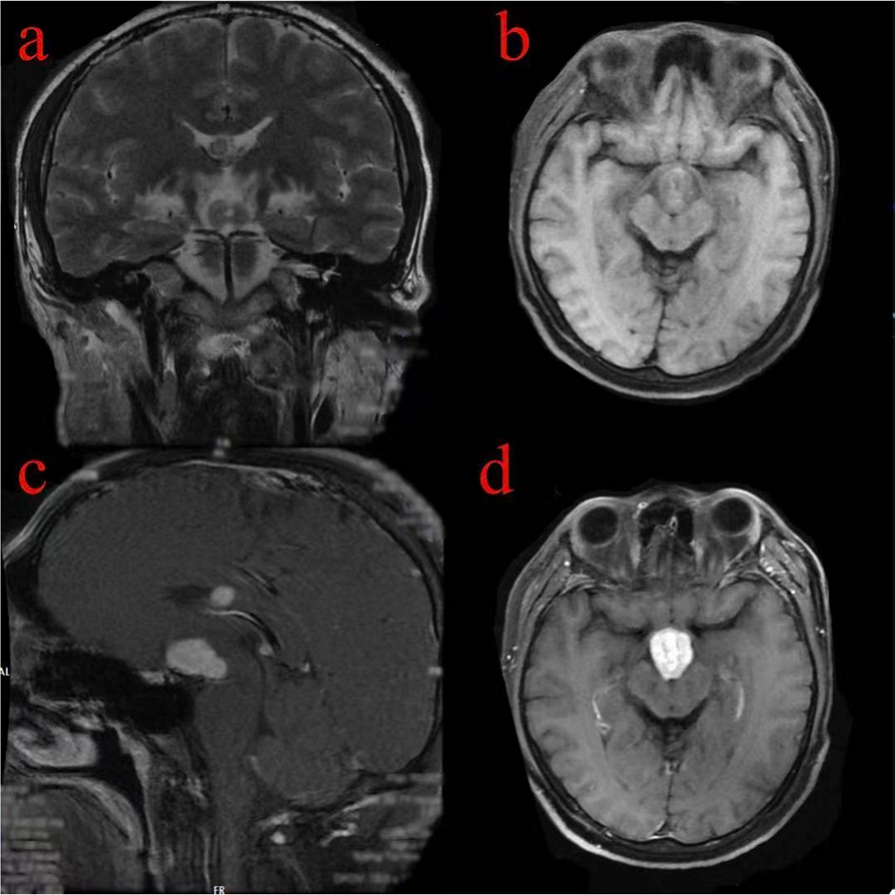
The lesions involving the lateral ventricles and the fourth ventricle show an isointense signal on axial T2WI (**a**), an isointense signal on axial T1WI (**b**), and homogeneous enhancement on sagittal and coronal T1c (**c** and **d**)

The addition of the PubMed database sourced 22 reported cases, with 35 lesions invading the lateral ventricle, 21 invading the fourth ventricle, and 11 invading the third ventricle (Figs. 2, 3, 4, and 5).

### Pathological findings

According to 2021 WHO classification [34], of the 29 identified cases of PCNSL in our institution, 26 (89.66%) were classified as DLBCL, 1 (3.45%) was chronic lymphocytic leukemia/small lymphocytic lymphoma, 1 (3.45%) was precursor B lymphoblastic leukemia/lymphoma, and 1 (3.45%) was high-grade B-cell lymphoma.

Of the 26 PCNSL-DLBCL cases, 12 (46.2%) were categorized as GCB, while the remaining 14 (53.8%) were classified as non-GCB. The median Ki-67 expression level for PCNSL involving the ventricle was found to be 80%, and BCL-2 was positive in 10 (45.45%) patients. There was no statistical difference between the expression of Ki-67 and BCL-2 in PCNSL with and without ventricular involvement. However, BCL-6 was positive in 64.29% of patients with ventricular involvement, which is lower than the positive rate of PCNSL without ventricular involvement (*P* = 0.022, 95% CI: 1.160–19.985). The clinicopathologic features of the 29 cases reviewed are summarized in Table 3.

### Therapy and perioperative complications

All patients included in this study underwent either surgery or stereotactic biopsy at Xiangya Hospital. Among the cases with ventricular involvement, 10 patients received total resection, 17 received partial or near-total resection, and 2 received stereotactic biopsy. In addition, 17 patients received chemotherapy, and 7 received radiotherapy (Table 2).

The occurrence rates of perioperative complications were compared between PCNSL and glioma involving the ventricle. The rates of intracranial infection, intracranial hemorrhage, thrombosis, and pulmonary infection were not found to be significantly different between the two groups (Table 5).

**Table 5:** Comparison of perioperative complications in PCNSL and glioma involving ventricles

Complications		PCNSL involving ventricles	Glioma involving ventricles	*P* value
Intracranial infection (*n*, %)	Yes	4 (13.79)	2 (6.90)	0.666
	No	25 (86.21)	27 (93.10)	
Intracranial hemorrhage (*n*, %)	Yes	1 (3.45)	2 (6.90)	1.000
	No	28 (96.55)	27 (93.10)	
Phlebothrombosis (*n*, %)	Yes	1 (3.45)	4(13.79)	0.349
	No	28 (96.55)	25(86.21)	
Pulmonary infection (*n*, %)	Yes	3 (10.34)	8 (27.59)	0.094
	No	26 (89.66)	21 (72.41)	
Ventriculo-peritoneal shunt (*n*, %)	Yes	3 (10.34)	2 (6.90)	1.000
	No	26 (89.66)	27 (93.10)	
Post-operative hydrocephalus (*n*, %)	Yes	5 (27.59)	8 (27.59)	0.345
	No	24 (82.76)	21 (72.41)	

### Prognosis and survival analysis

Among the 29 newly diagnosed patients with ventricular PCNSL, prognostic follow-up data were available for 23 cases (ranging from 0 to 120 months). In the non-ventricular PCNSL group, follow-up information was available for 24 out of 29 patients (ranging from 0 to 133 month). There was no significant difference in survival time (log-rank *P* = 0.245) between the two groups. Moreover, the subtype of PCNSL-DLBCL (GCB vs. non-GCB) did not affect OS (log-rank *P* = 0.290).

In terms of prognostic factors, our study found that gender and age were significant predictors of PCNSL involving ventricles. Male patients had a longer survival time than female patients (log-rank *p* = 0.025), and age above 60 years was a poor prognostic factor (log-rank *p* = 0.042). However, the number of lesions (single or multiple) (log-rank *p* = 0.827), the surgical treatment method (log-rank p = 0.619), chemotherapy (log-rank *p* = 0.138), radiotherapy (log-rank *p* = 0.85), the expression of BCL-6 (log-rank *p* = 0.89), and BCL-2 (log-rank *p* = 0.352)were not significant prognostic factors for PCNSL involving ventricles.

Our study also found no significant difference in survival outcomes between patients who received chemotherapy and those who did not. For ventricular cases, the 3-month and 3-year overall survival rates for patients who received chemotherapy were 93.8% and 25%, respectively. Meanwhile, the 3-month and 3-year overall survival rates for patients who did not receive chemotherapy were 60% and 0%, respectively.

## Discussion

In this paper, we present a retrospective evaluation of 29 new cases of PCNSL with ventricular involvement from Xiangya Hospital, along with a review of 22 previously reported cases from the PubMed database. We analyzed the clinical characteristics, pathology, radiology, and treatment of PCNSL involving ventricles, and compared them with PCNSL cases not involving ventricles, and glioma cases involving ventricles. This study provides new insights into the clinical behavior of intraventricular PCNSL and reinforces its distinction from other intraventricular tumors. By identifying similarities and differences with non-intraventricular PCNSL, our findings contribute to a more nuanced understanding of this rare entity. Our findings offer new insights into the unique clinical behavior of ventricular PCNSL. The distinct imaging variability on T2-weighted MRI, frequent multifocal presentation (40.91%), and lower BCL-6 expression.

In our series of patients with PCNSL involving ventricles, the median age of diagnosis was 52 years, which is somewhat lower than that reported in most previous studies [35–37]. The male-to-female ratio is 1.68:1, which is consistent with the predilection toward males reported in the majority of retrospective series [37, 38]. Signs of increased intracranial pressure leading to headaches were common (47.06%), while seizures were uncommon (7.84%). Patients with primary central nervous system lymphoma (PCNSL) involving the ventricle are more likely to undergo surgical removal instead of biopsy, as malignant neoplasms invading the ventricles are often misdiagnosed as gliomas. Additionally, patients whose lesions involve the ventricles are more prone to developing life-threatening hydrocephalus, which must be treated surgically.

Regarding the ventricular PCNSL imaging presentation, all lesions were hypointense to isointense on T1WI. However, T2WI encompassed all three signals, namely hypointense, isointense, and hyperintense. This shows that T2WI characteristics can be more variable than those of T1WI. All lesions demonstrated homogeneous or heterogeneous enhancement and varying degrees of edema. Thirteen cases (59.09%) exhibited a solitary lesion, while nine cases (40.91%) presented with multiple lesions, suggesting that multifocal involvement is not uncommon in ventricular PCNSL. These imaging findings were identical to those of previous PCNSL cases [39–41].

Of the PCNSL-DLBCL cases, twelve were of the GCB subtype, and the remaining 14 were of the non-GCB type, but there was no significant difference in prognosis between the two groups. However, positive immunoreactivity with BCL-6 was found to be lower in patients with PCNSL involving ventricles than in those without ventricular involvement (Table 3), which is a novel finding not previously reported in the literature.

Although our study found no significant difference in prognosis between the two groups, we observed that two patients with PCNSL involving ventricles died due to chemotherapy-related infection, or operative factors, which could have potentially impacted the analysis of prognosis. We went ahead and compared the chemotherapy and no-chemotherapy groups using Fisher’s exact probability test. Furthermore, the small sample size and variability in treatment received limit the Generalizability of our results. It is worth noting that one patient who did not receive chemotherapy had a good prognosis, surviving for more than 21 months at the last follow-up. In contrast, only one patient who received chemotherapy and autologous stem cell transplantation died 5 months after surgery. However, Haegelen [8] reported that one patient who received chemotherapy followed by autologous stem cell transplantation and entire brain radiotherapy was still alive without recurrence 7 months later. These findings portend the need for further investigation into the efficacy of chemotherapy and autologous stem cell transplantation for PCNSL involving ventricles.

Our study did not find significant differences in perioperative complications between PCNSL and glioma. This implies that the surgical risks for both diseases are similar. However, larger multicenter studies are needed to precisely summarize the clinical manifestations, imaging features, surgical treatments, and molecular predictors of outcomes to individualize the treatment options due to the rarity of this disease.

Limitations of this study include the retrospective design, small sample size, variable treatment regimens, and potential selection bias from literature-derived cases. Larger, prospective, multicenter studies are needed to confirm our findings and to better define the imaging characteristics, clinical behavior, and optimal therapeutic strategies for this rare but clinically distinct subtype of PCNSL.

## Conclusion

This paper presents a case series examining the clinical presentation of PCNSL involving ventricles. Although our study found a lower expression of BCL-6 in the ventricular group compared to the non-ventricular group, larger sample sizes are necessary to confirm its reliability and to explore the underlying reasons for the reduced BCL-6 expression in ventricular PCNSL. It is imperative to investigate the potential causes of tumorigenesis in these patients. Based on our current findings, we recommend that treatment for PCNSL involving the ventricle should follow the same protocol as for PCNSL in general, which involves surgical removal or biopsy followed by a combination of chemotherapy using high-dose MTX, and other therapies, depending on the patient’s specific case [42].

## Data Availability

The datasets used and/or analyzed during the current study are available from the corresponding author on reasonable request.

## Abbreviations

PCNSL: Primary central nervous system lymphoma
CNS: Central nervous system
T1WI: T1-weighted MRI
T2WI: T2-weighted MRI
DLBCL: Diffuse large B-cell lymphoma
OS: Overall survival
BCL: B-cell lymphoma
GFAP: Glial fibrillary acidic protein
MUM-1: Multiple myeloma oncogene 1
EBER: Epstein-Barr virus small RNA
GCB: Germinal center B-cell
